# Overexpression of *STARCH BRANCHING ENZYME II* increases short-chain branching of amylopectin and alters the physicochemical properties of starch from potato tuber

**DOI:** 10.1186/s12896-015-0143-y

**Published:** 2015-04-29

**Authors:** David A Brummell, Lyn M Watson, Jun Zhou, Marian J McKenzie, Ian C Hallett, Lyall Simmons, Margaret Carpenter, Gail M Timmerman-Vaughan

**Affiliations:** The New Zealand Institute for Plant & Food Research Limited, Food Industry Science Centre, Private Bag 11600, Palmerston North, 4442 New Zealand; The New Zealand Institute for Plant & Food Research Limited, Mount Albert Research Centre, Private Bag 92169, Auckland, 1142 New Zealand; The New Zealand Institute for Plant & Food Research Limited, Canterbury Agriculture & Science Centre, Private Bag 4704, Christchurch, 8140 New Zealand

**Keywords:** Amylopectin branching, *GBSS* silencing, *SBEII* overexpression, Starch gelatinisation, Starch properties, *Solanum tuberosum*

## Abstract

**Background:**

Starch is biosynthesised by a complex of enzymes including various starch synthases and starch branching and debranching enzymes, amongst others. The role of all these enzymes has been investigated using gene silencing or genetic knockouts, but there are few examples of overexpression due to the problems of either cloning large genomic fragments or the toxicity of functional cDNAs to bacteria during cloning. The aim of this study was to investigate the function of potato *STARCH BRANCHING ENZYME II* (*SBEII*) using overexpression in potato tubers.

**Results:**

A hybrid *SBEII* intragene consisting of potato cDNA containing a fragment of potato genomic DNA that included a single intron was used in order to prevent bacterial translation during cloning. A population of 20 transgenic potato plants exhibiting *SBEII* overexpression was generated. Compared with wild-type, starch from these tubers possessed an increased degree of amylopectin branching, with more short chains of degree of polymerisation (DP) 6–12 and particularly of DP6. Transgenic lines expressing a *GRANULE-BOUND STARCH SYNTHASE* (*GBSS*) RNAi construct were also generated for comparison and exhibited post-transcriptional gene silencing of *GBSS* and reduced amylose content in the starch. Both transgenic modifications did not affect granule morphology but reduced starch peak viscosity. In starch from *SBEII*-overexpressing lines, the increased ratio of short to long amylopectin branches facilitated gelatinisation, which occurred at a reduced temperature (by up to 3°C) or lower urea concentration. In contrast, silencing of *GBSS* increased the gelatinisation temperature by 4°C, and starch required a higher urea concentration for gelatinisation. In lines with a range of *SBEII* overexpression, the magnitude of the increase in SBEII activity, reduction in onset of gelatinisation temperature and increase in starch swollen pellet volume were highly correlated, consistent with reports that starch swelling is greatly dependent upon the amylopectin branching pattern.

**Conclusion:**

This work reports the first time that overexpression of *SBEII* has been achieved in a non-cereal plant. The data show that overexpression of *SBEII* using a simple single-intron hybrid intragene is an effective way to modify potato starch physicochemical properties, and indicate that an increased ratio of short to long amylopectin branches produces commercially beneficial changes in starch properties such as reduced gelatinisation temperature, reduced viscosity and increased swelling volume.

**Electronic supplementary material:**

The online version of this article (doi:10.1186/s12896-015-0143-y) contains supplementary material, which is available to authorized users.

## Background

Starch serves as the most important carbohydrate storage reserve for plants, and provides the foundation for human and animal nutrition as well as a raw material for numerous food and industrial applications [1]. Starch consists of two molecules, long essentially linear chains of α-(1 → 4)-linked glucopyranosyl residues (amylose), and a much larger, highly branched molecule (amylopectin) consisting of thousands of short α-(1 → 4)-linked glucan chains of degree of polymerisation (DP) 6 to >100 that are attached by α-(1 → 6)-linked branch points [2].

Amylose and amylopectin are synthesised in the plastids, where they assemble into a semicrystalline granule. Amylopectin is the major component, making up 70–80% of starch in most species. Amylose is synthesised by granule-bound starch synthase (GBSS), whereas a large complex of enzymes is required to synthesise amylopectin. This complex consists of four soluble starch synthases (SSI, SSII, SSIII, SSIV) and two types of starch branching enzyme (SBEI, SBEII), with various debranching enzymes, kinases and other enzymes also involved [2-4]. The availability of various mutants lacking particular enzyme activities has shown how the properties of starch from different species are altered by changes to composition or structure. For example, starch from the *waxy* mutant of maize (*Zea mays* L.) (deficient in GBSS and virtually amylose-free) had a lower gelatinisation temperature, whereas that from wheat (*Triticum aestivum* L.) had an unaltered gelatinisation temperature but altered pasting properties [5]. Starch from potato (*Solanum tuberosum* L.) where *GBSS* had been down-regulated produced gels with improved stability and clarity [6].

Differences in amylopectin branching affect granule crystallinity, which together with differences between species in granule size and shape result in altered thermal, pasting and biophysical properties [7,8]. Amylopectin branching is carried out by SBEI and SBEII, enzymes that create branch points by cleaving α-(1 → 4)-linkages and reattaching the glucan chain via an α-(1 → 6)-linkage. Alterations in SBE activity affect the number and size distribution of amylopectin branches. In cereals, there are three isoforms of SBE: SBEIa, SBEIIa and SBEIIb, and in the *amylose extender* mutant of maize that lacks SBEIIb, amylopectin had fewer branches and a higher proportion of longer chains [7] that are essentially amylose. Similarly in rice (*Oryza sativa* L.), down-regulation of *SBEIIb* decreased the proportion of short branches and increased the proportion of intermediate and long branches [9]. In barley (*Hordeum vulgare* L.), only when both *SBEIIa* and *SBEIIb* were down-regulated was a significant reduction in the frequency of amylopectin branches observed [10]. Indeed, down-regulation of all three isoforms of SBE in barley produced starch that was entirely amylose [11]. *In vitro* experiments with rice enzymes found that SBEIIb transferred chains of DP6 and DP7, SBEIIa transferred chains of DP6–15, and SBEIa produced a range of short and intermediate chains of DP ≤ 40 [12].

In potato only two isoforms of SBE are involved, SBEI (also known as SBE B) and SBEII (also known as SBE A) [13,14]. SBEI is the major isoform, but its down-regulation had little effect on starch structure [15,16]. Knockdown of the minor SBEII isoform, however, increased the average chain length of amylopectin, which, as in the maize *amylose extender* mutant [17], resulted in an increase in apparent amylose content due to the increased proportion of longer linear amylopectin chains [14]. Knockdown of both *SBEI* and *SBEII* produced potato starch that was >70% apparent amylose and had a very high phosphorus content [18].

Overexpression of SBE activity to modify starch structure is a much more difficult proposition than knockdown, due to the enzyme’s deleterious effects on bacteria during cloning of the constructs [14]. Overexpression of bacterial glycogen synthase or glycogen branching enzyme produced starch with more branches and more short chains [19-21], but only one study has successfully overexpressed a plant SBE enzyme. This involved the cloning of an 18 kb genomic fragment containing the native promoter and coding region, including 21 introns, of the rice *SBEIIb* gene, and expressing it in a rice *SBEIIb*-deficient background [22]. We aimed to examine the role of potato SBEII in starch branching and resultant effects on potato starch properties. Potato is now the third most important food crop in the world and the most important non-cereal [23], whose starch also has many uses in the food and manufacturing industries [1] due to its advantages over cereal starches (see Discussion). To do this we employed a simple hybrid cDNA/gDNA intragene construct containing a single intron to prevent bacterial translation, expressed under the control of the strong tuber-specific potato *GBSS* promoter. An additional way to increase the proportion of branched amylopectin in the granule is to down-regulate *GBSS* and reduce amylose content, so knockdown *GBSS* lines were also generated for comparison.

## Results

### Characterisation of transgenic potato plants

**Figure 1:**
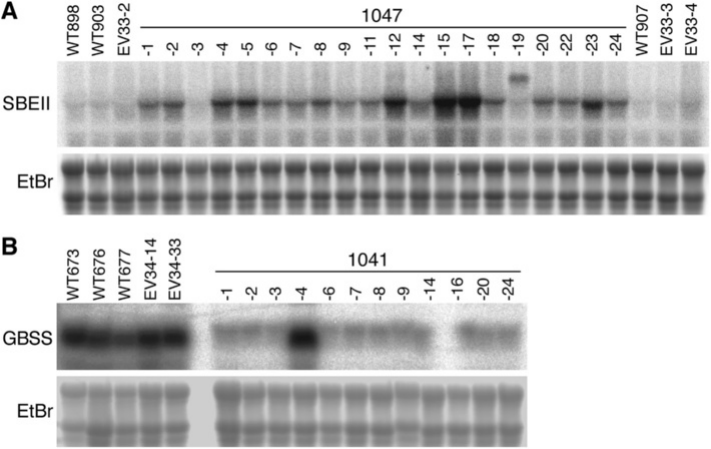
RNA gel blots showing alterations to *SBEII* and *GBSS* mRNA abundance in transgenic plants. **(A)** *SBEII* mRNA abundance in tubers from plants transformed with a *SBEII* overexpression construct (1047 lines). **(B)** *GBSS* mRNA abundance in tubers from plants transformed with a *GBSS* RNAi construct (1041 lines). Wild-type (WT) and empty vector transformants (EV) are shown for comparison. Ethidium bromide (EtBr) staining of RNA (with contrast reversed) was used to confirm the equivalency of loading.

Initial experiments confirmed an earlier report that a complete cDNA of potato *SBEII* was unable to be propagated in *Escherichia coli* [14]. Despite the use of several different bacterial strains, very few colonies were produced and all were subsequently discovered to contain frame-shift mutations (data not shown). Therefore, a complete but hybrid *SBEII* intragene containing a single intron to prevent bacterial translation was assembled from cDNA and genomic DNA fragments (See Methods and Additional file 1: Figure S1). A population of 28 plants transformed with the *SBEII-intron* intragene under the control of the *GBSS* promoter was generated (Additional file 1: Figure S2). Of these, 20 plantlets showing good root development were grown to maturity, and tubers exhibited a range of *SBEII* mRNA abundances (Figure 1A). *SBEII* mRNA accumulation in these lines ranged from less than wild-type controls (line 1047–3), moderate overexpression (lines 1047–1, 2, 4, 5, 6, 7, 8, 9, 11, 14, 18, 20, 22, 24) to high overexpression (lines 1047–12, 15, 17, 23). Line 1047–19 exhibited approximately wild-type mRNA abundance of *SBEII* but with an additional anomalous larger *SBEII* transcript presumably derived from an aberrant insertion event. To confirm that mRNA accumulation of the structurally unrelated starch synthase enzymes SSII and SSIII was not affected by manipulation of *SBEII* expression, the same gel blot was stripped and re-hybridised. Sequential re-hybridisation with labelled probes for *SSII* and *SSIII* showed that the mRNA abundances of these two genes were essentially indistinguishable from wild-type and empty vector controls (Additional file 1: Figure S3).

Of a population of 24 plants putatively transformed with a *GBSS* inverted repeat transgene, only 12 were positive for the presence of both the transgene and the selectable marker gene (Additional file 1: Figure S2). Of these 12 lines, 11 showed a strong reduction in *GBSS* mRNA accumulation (Figure 1B). One line, 1041–4, did not show any apparent posttranscriptional gene silencing, and this line was retained as an additional control for some tests since it was transgenic but *GBSS* mRNA accumulation was not reduced relative to wild-type.

**Figure 2:**
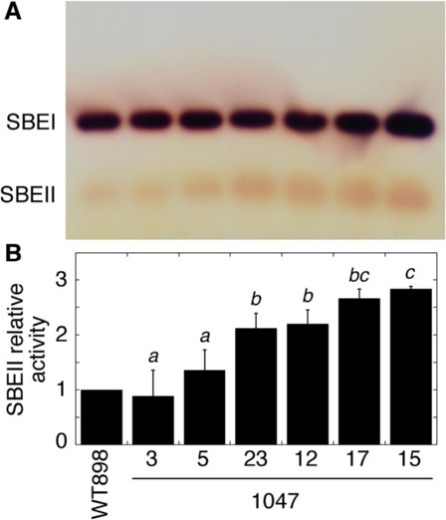
SBE activity in tubers from a series of lines transformed with a *SBEII* overexpression construct. **(A)** Native PAGE activity gel with SBE activity visualized by iodine staining. **(B)** Mean relative SBEII activity ± SD as determined from the density of the spots. Data are from technical replicate protein preparations from the same pooled tissue as Figure 1 run on different gels on different days (n = 3). Means not sharing a common letter are significantly different between groups at *P* = 0.05 as determined by LSD after a one-way ANOVA test.

To assess the effect of overexpression of a *SBEII* intragene on SBE enzyme activity, SBEII was separated from the major and predominant SBEI by separating proteins using native polyacrylamide gel electrophoresis (PAGE), followed by an in-gel activity reaction and staining for SBE reaction products (Figure 2A). The two isoforms of SBE were identified based on the migration of rice and wheat enzymes [17,22,24] and the relative activities of potato SBEI and SBEII [14]. SBEII activity approximately reflected the abundance of *SBEII* mRNA, being similar to wild-type in line 1047–3, and with a range of activities greater than wild-type in a selection of other lines (WT < 1047–5 < 1047–23 = 1047–12 ≤ 1047–17 ≤ 1047–15). The greatest enhancement was in lines 1047–15 and 17, which possessed SBEII activity almost 3-fold that of wild-type (Figure 2B).

### Effects of altered *GBSS* and *SBEII* expression on the structure of starch

The effectiveness of silencing of *GBSS* was determined by measuring the percentage of amylose present in the starch from these plants (Table 1). Wild-type and empty vector control starches were approximately 19–26% amylose, but this was reduced to 3.1–5.6% in lines 1041–1, 3 and 16. In line 1041–4, which showed no reduction in *GBSS* mRNA abundance relative to controls (Figure 1B), amylose comprised 21.3% of the total starch, a content within the range found in controls. The percentage of amylose in the 1047 lines overexpressing *SBEII* ranged from 17.0 to 23.4%, and was similar to controls.

**Table 1:** **Amylose content in starch from potato tubers of a range of lines exhibiting silencing of** ***GBSS*** **(1041 lines) or overexpressing** ***SBEII*** **(1047 lines)**

**Line**	**% Amylose**
WT676	26.3 ± 0.3
WT898	21.4 ± 0.3
WT903	21.5 ± 0.9
EV33-3	20.6 ± 0.2
EV33-5	18.8 ± 0.8
EV34-33	22.2 ± 0.2
1041-1	4.9 ± 0.3
1041-3	3.1 ± 1.0
1041-4	21.3 ± 0.2
1041-16	5.6 ± 0.3
1047-3	19.2 ± 0.3
1047-5	17.0 ± 0.3
1047-12	17.9 ± 0.6
1047-15	23.4 ± 0.8
1047-17	19.4 ± 0.3
1047-23	22.2 ± 0.8

Starch was also prepared and assayed from wild-type (WT) and empty vector (EV) control plants grown alongside the transgenic lines. Data are means ± SD (n = 4 technical replicates).

**Figure 3:**
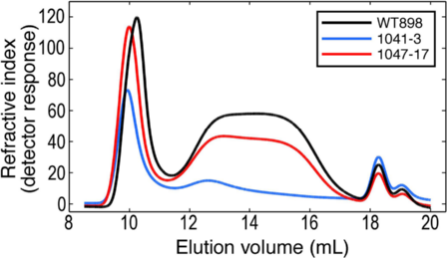
Size separation of tuber starch polyglucans. Starch was solubilised in NaOH and gelatinised by heat before size exclusion chromatography on a Waters Ultrahydrogel Linear column. Curves are the means of measurements on three independent biological replicates per line. Carbohydrate in the eluate was measured by refractive index.

Size exclusion chromatography was used to separate amylose from amylopectin (Figure 3). Elution profiles consisted of two main regions, a sharp peak eluting at 9–11 min representing high molecular weight amylopectin, and a broader more disperse lower molecular weight peak eluting at 12–17 min representing amylose [25-27]. The small peaks eluting at 18–19 min were probably due to free sugars. The relative areas of the amylopectin and amylose peaks showed that suppression of *GBSS* in line 1041–3 substantially reduced the content of amylose and that overexpression of *SBEII* in line 1047–17 slightly reduced the content of amylose, consistent with the biochemical assays (Table 1). The amylopectin peaks for both transgenic lines eluted at slightly higher molecular weight than wild-type.

**Figure 4:**
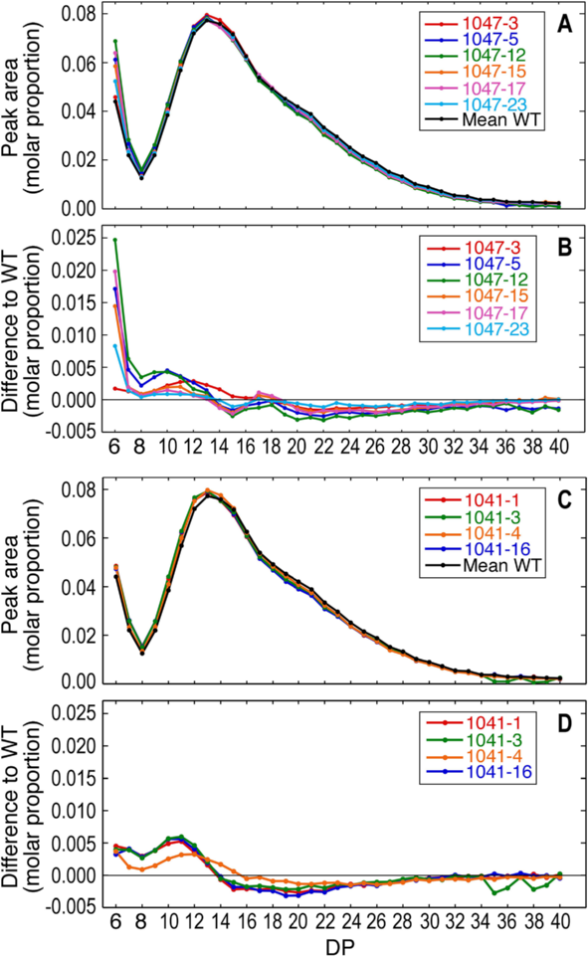
Chain length distribution of amylopectin unit chains. **(A)** Comparison of size distribution of amylopectin unit chains in starch from tubers with altered *SBEII* expression. **(B)** Difference plot showing changes in chain length distribution relative to wild-type. **(C)** Comparison of size distribution of amylopectin unit chains in starch from tubers with altered *GBSS* expression. **(D)** Difference plot showing changes in chain length distribution relative to wild-type.

A modified fluorophore-assisted carbohydrate electrophoresis (FACE) method was used to examine the effects of altered *SBEII* and *GBSS* expression on the chain length distribution of debranched polyglucans (Figure 4). Polyglucans from wild-type showed a moderate number of side chains of DP6, few side-chains of DP7-9, and a predominant peak of side chains spanning the DP ~ 10-24 range (Figure 4A, C). The number of side chains of DP ≥ 37 was small. Overexpression of *SBEII* in various lines had little effect on the chain length distribution of moderately sized chains of DP13-18, slightly reduced the abundance of larger chains of DP ≥ 20 and increased the number of short chains of DP ≤ 12 (Figure 4B). The greatest effect was on the number of chains of DP6, with the increase in lines 1047–5, 12, 15, 17 and 23 ranging from 19 to 56% greater than wild-type (Figure 4A). However, there was no obvious correlation between the increase in DP6 chain abundance and relative SBEII activity. The only line in which an increase in the abundance of DP6 side chains was not observed was line 1047–3 (Figure 4B), which was the line in which SBEII mRNA abundance and activity were not increased (Figures 1 and 2).

In transgenic lines with a higher proportion of amylopectin in the granule due to reduced amylose content (lines 1041–1, 3, 16), the chain length distribution of polyglucans was very similar to wild-type (Figure 4C). There was a small increase in the number of side chains of DP6-13, and a small decrease in those of DP15-28 (Figure 4D), changes that were much less pronounced in line 1041–4 that did not exhibit silencing of *GBSS* (Figure 1). This suggests that the reduced activity of GBSS not only increased the proportion of amylopectin in starch (by reducing the biosynthesis of amylose), but also had some small effects on its structure.

Amylopectin chain length distribution and the phosphorus content of the starch are related, with phosphorylation predominantly occurring on longer amylopectin chains [28]. Many studies of starch phosphorylation underestimate the true abundance of phosphate groups by measuring only C6-linked phosphates, and ignoring the more acid-labile C3 linkage. We have used an improved method that allows determination of phosphate groups with both linkages. The ratio of C6 phosphate content to C3 phosphate content was ~4.5:1 in all lines tested (Table 2). Total phosphate content was ~40 nmol per mg starch in a wild-type line, an empty vector transformant and two lines with knockdown of *GBSS* expression. However, total phosphate content was reduced by ~25% in two lines in which *SBEII* expression was increased, with both C6- and C3-linked phosphate content being reduced.

**Table 2:** **Phosphate content of tuber starches from lines exhibiting silencing of** ***GBSS*** **(1041 lines) or overexpressing** ***SBEII*** **(1047 lines)**

**Line**	**C6 PO**_**4**_	**C3 PO**_**4**_	**Total**
WT898	32.8 ± 1.8	7.5 ± 0.6	40.3
EV33-5	29.0 ± 2.7	6.4 ± 0.6	35.4
1041-3	35.2 ± 6.1	8.8 ± 1.9	44.0
1041-16	31.7 ± 1.6	7.1 ± 0.5	38.8
1047-15	24.1 ± 2.5	5.1 ± 0.3	29.1
1047-17	24.5 ± 2.1	5.8 ± 0.1	30.2

WT: wild-type; EV: empty vector. Data are mean nmol Glc6P per mg starch ± SD and mean nmol Glc3P per mg starch ± SD (n = 3 biological replicates). Total nmol phosphate per mg starch was calculated as the sum of C6 plus C3 phosphates.

### Effects of altered *GBSS* and *SBEII* expression on the morphology of starch granules

**Figure 5:**
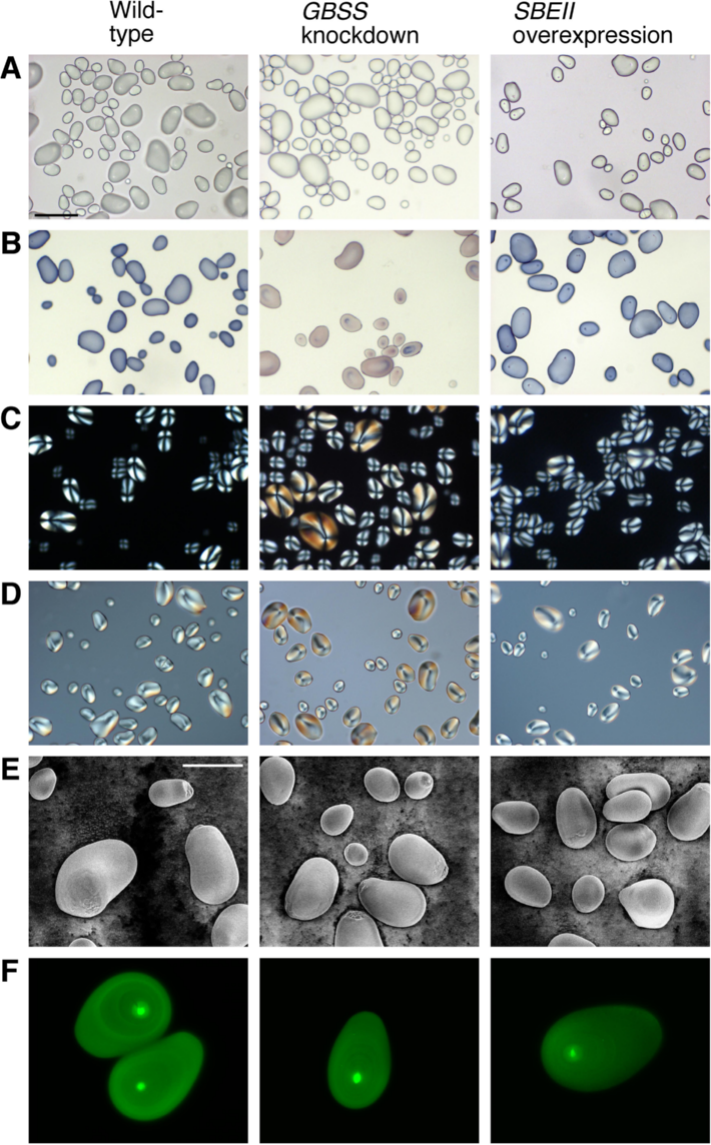
Images of tuber starch granules from wild-type and transgenically modified potato tubers. **(A)** Brightfield. **(B)** Brightfield, starch stained with I_2_/KI. **(C)** Polarised light. **(D)** Differential interference contrast. **(E)** Variable pressure scanning electron microscopy. **(F)** Optical section of starch fluorescently labelled with APTS taken using confocal scanning laser microscopy. Lines used were WT898, 1041–3 and 1047–17. The scale bar (in panel **A**) for light microscopy pictures **(A–D)** represents 100 μm, and for the scanning electron microscopy **(E)** represents 40 μm.

To test whether the morphology of the starch granules was altered by the transgenic modifications, a range of microscopy techniques was employed (Figure 5). Brightfield microscopy showed a wide range of granule sizes in wild-type starch (Figure 5A), and neither knockdown of *GBSS* nor overexpression of *SBEII* had any obvious effect on the size range distribution of the granules. Granules from wild-type and *SBEII* overexpressors stained dark blue with iodine (Figure 5B), whereas granules from *GBSS* knockdown plants showed the pale purple colour typical of a low amylose content but with visible dark blue staining at the hilum core. Examination under polarised light detected birefringence visible as a ‘Maltese cross’ shape and blue and yellow sectors (Figure 5C). Birefringence was higher in the *GBSS* knockdown line, presumably since these lines are higher in amylopectin content, which has a more ordered orientation (crystallinity) than amylose. An increased birefringence in the *GBSS* knockdown line was also observed using differential interference contrast microscopy (Figure 5D). Variable pressure scanning electron microscopy showed that the external morphology of the starch granules was not affected by *GBSS* knockdown or by *SBEII* overexpression (Figure 5E).

Details of the internal structure of the granules were examined by fluorescent labelling with 8-amino-1,3,6-pyrenetrisulfonic acid (APTS) combined with confocal laser scanning microscopy (Figure 5F). APTS labels the single reducing end of each molecule with high efficiency, and since amylose molecules are much smaller than the highly branched amylopectin molecules, they exhibit a higher fluorescence (on a fluorescently-labelled reducing end per total Glc residue basis). A small intensely labelled region at the hilum core was visible in all the lines, indicating a high concentration of amylose at this site. This was observed even in the low amylose *GBSS* knockdown line, and is consistent with the iodine staining of this site seen under brightfield microscopy. Growth rings were more visible in the wild-type, suggesting that the deposition of amorphous and crystalline growth rings may have been altered in the transgenic lines. However, overall fluorescence was lower in both of the transgenic lines, and was too low for clear visualisation of growth rings in the *SBEII* overexpressors.

### Thermal and gelatinisation properties of transgenically modified starch

The different contents of amylose, short-branched amylopectin and phosphorus in the starch would be expected to result in altered thermal and gelatinisation properties. Both knockdown of *GBSS* expression and overexpression of *SBEII* markedly altered the onset of gelatinisation temperature (*T*_o_), but in opposite ways (Table 3). In wild-type and empty vector controls, *T*_o_ ranged from 54.2 to 55.4°C, whereas in three lines with reduced *GBSS* expression and lowered amylose content (1041–1, 3 and 16), *T*_o_ was increased by ~4°C, to 59.3 to 60.0°C. In the transgenic line 1041–4, with unaltered *GBSS* expression, *T*_o_ was not increased relative to controls. In contrast, overexpression of *SBEII* in various 1047 lines reduced *T*_o_ by up to 3°C (Table 3). In these lines, a trend towards lowered *T*_o_ (WT > 1047–5 = 1047–23 > 1047–12 ≥ 1047–15 ≥ 1047–17) correlated strongly (*r* = −0.931) with the trend of increasing SBEII activity (Figure 2). In line 1047–3, which showed SBEII activity similar to wild-type, *T*_o_ was not reduced and was within the range of variability seen in control lines. The temperature of gelatinisation peak (*T*_p_), temperature of gelatinisation conclusion (*T*_c_) and gelatinisation enthalpy (Δ*H)* showed similar trends to *T*_o_ in all of the lines.

**Table 3:** **Thermal properties of tuber starch granules from plants exhibiting silencing of** ***GBSS*** **(1041 lines) or overexpressing** ***SBEII*** **(1047 lines)**

**Line**	***T***_**o**_ **(°C)**	***T***_**p**_ **(°C)**	***T***_**c**_ **(°C)**	**Δ** ***H*** **(J g** ^**−1**^ **)**
WT676	54.7 ± 0.7^*e*^	58.1 ± 0.4^*ef*^	61.6 ± 0.0^*d*^	15.4 ± 0.4^*bcde*^
WT898	55.3 ± 0.2^*e*^	58.5 ± 0.1^*ef*^	62.1 ± 0.1^*d*^	15.9 ± 0.2^*bcde*^
WT903	55.4 ± 0.6^*e*^	58.6 ± 0.3^*ef*^	61.6 ± 0.0^*d*^	15.7 ± 0.1^*bcde*^
EV33-3	54.2 ± 0.7^*cd*^	57.7 ± 0.4^*d*^	61.6 ± 0.5^*d*^	15.8 ± 2.7^*bcde*^
EV33-5	55.4 ± 0.6^*e*^	58.7 ± 0.4^*fg*^	62.1 ± 0.1^*d*^	15.0 ± 0.5^*abcd*^
EV34-33	54.9 ± 0.2^*de*^	58.2 ± 0.1^*de*^	61.7 ± 0.0^*d*^	14.9 ± 0.5^*abc*^
1041-1	59.3 ± 0.1^*f*^	63.1 ± 0.2^*h*^	67.5 ± 0.1^*f*^	18.2 ± 0.1^*f*^
1041-3	59.6 ± 0.6^*f*^	63.8 ± 0.4^*i*^	68.7 ± 0.3^*g*^	17.2 ± 1.5^*ef*^
1041-4	53.8 ± 0.5^*c*^	57.8 ± 0.2^*d*^	61.9 ± 0.6^*d*^	16.8 ± 1.7^*def*^
1041-16	60.0 ± 0.4^*f*^	63.4 ± 0.3^*hi*^	67.9 ± 0.3^*f*^	17.9 ± 0.4^*f*^
1047-3	55.2 ± 0.3^*e*^	59.1 ± 0.5^*g*^	64.9 ± 1.2^*e*^	16.6 ± 1.5^*cdef*^
1047-5	53.6 ± 0.8^*c*^	57.0 ± 0.7^*c*^	60.9 ± 0.1^*c*^	14.2 ± 1.6^*ab*^
1047-12	52.8 ± 0.9^*b*^	55.9 ± 0.5^*b*^	59.1 ± 0.2^*ab*^	13.7 ± 0.5^*a*^
1047-15	52.3 ± 0.4^*ab*^	55.6 ± 0.2^*b*^	59.2 ± 0.2^*b*^	14.5 ± 0.5^*ab*^
1047-17	52.0 ± 0.3^*a*^	55.0 ± 0.1^*a*^	58.5 ± 0.2^*a*^	14.6 ± 0.4^*ab*^
1047-23	53.7 ± 0.2^*c*^	56.7 ± 0.2^*c*^	59.7 ± 0.3^*b*^	15.0 ± 1.3^*abcd*^

WT: wild-type; EV: empty vector. Data are means ± SD (n = 4 biological replicates). Means in the same column not sharing a common letter are significantly different between groups at *P* = 0.05 as determined by LSD after a one-way ANOVA test.

*T*_o_ = temperature of onset of gelatinisation peak.

*T*_p_ = temperature of gelatinisation peak.

*T*_c_ = temperature of gelatinisation conclusion.

Δ*H* = gelatinisation enthalpy of starch.

**Figure 6:**
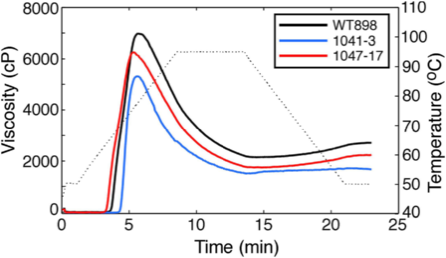
Comparison of pasting properties of tuber starch from wild-type and transgenic lines. Pasting properties were assessed in starch from lines exhibiting *GBSS* knockdown (1041–3) or *SBEII* overexpression (1047–17). Changes in viscosity of 7% (w/w) starch suspensions during heating and subsequent cooling were measured by RVA. Curves are the means of measurements on three independent biological replicates per line. The thin dotted line shows the change in temperature during measurement.

An examination of starch pasting properties using a rapid visco analyser (RVA) detected differences between the lines (Figure 6), including the lowered *T*_o_ of starch from line 1047–17 and the increased *T*_o_ of starch from line 1041–3. Other than the onset temperature, pasting profiles were similar in shape, but with a reduction in peak viscosity of ~10% for line 1047–17 and ~24% for line 1041–3. Final viscosities were also reduced in both transgenic lines.

**Figure 7:**
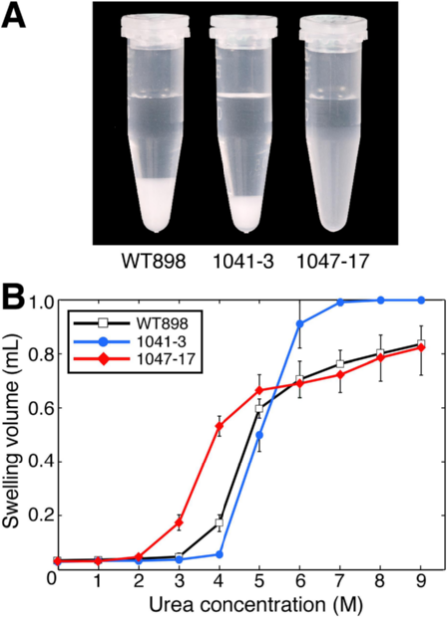
Changes in gelatinisation of tuber starch granules from transgenic potato lines. **(A)** Effect of *GBSS* knockdown (1041–3) or *SBEII* overexpression (1047–17) on starch granule swelling in 4 M urea. **(B)** Comparison of swelling of starch granules in a range of urea concentrations. Gelatinisation was assessed by measuring the volume of the swollen starch pellet. Data are mean pellet volumes ± SD (n = 4 biological replicates).

The greatest difference in physicochemical properties was seen in the gelatinisation of starch granules in urea. In 4 M urea, starch granules from line 1041–3 showed very little gelatinisation, with a swollen pellet volume only one-third that of wild-type starch granules (Figure 7A). Starch from lines 1041–1 and 1041–16 produced similar results (data not shown). In contrast, starch granules from line 1047–17 exhibited a huge amount of gelatinisation, producing a large and diffuse pellet with a swollen volume 3-fold greater than that of wild-type. Starch from line 1047–15 produced similar results (data not shown). Examination of a range of urea concentrations showed that gelatinisation was initiated at a lower urea concentration in starch granules from 1047–17, but followed a similar curve to wild-type and achieved a similar maximum swollen volume (Figure 7B). Gelatinisation followed a very different pattern in starch granules from 1041–3, beginning at a higher urea concentration and showing a remarkably steep increase in swollen pellet volume over a change in urea concentration from 4 M to 6 M. The maximum swelling volume achieved by 1041–3 was up to 25% greater than wild-type, and at urea concentrations above 7 M occupied the entire 1 mL volume of the original liquid provided.

**Figure 8:**
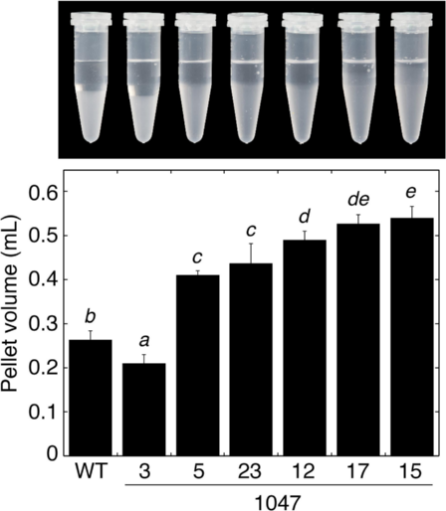
Effects of altered *SBEII* expression on the gelatinisation of tuber starch granules in 4 M urea. Data are mean pellet volumes ± SD (n = 3 biological replicates). Means not sharing a common letter are significantly different between groups at *P* = 0.05 as determined by LSD after a one-way ANOVA test. A photograph of one of the replicates is shown above the histogram.

The extent of *SBEII* overexpression had a clear effect on the swollen volume of the starch granules in 4 M urea (Figure 8). In line 1047–3, swollen pellet volume was less than wild-type. However, the other lines formed a series of increasing swollen pellet volume, in the order WT < 1047–5 = 1047–23 < 1047–12 ≤ 1047–17 ≤ 1047–15. This order reflected the trend in increasing SBEII activity (Figure 2), confirming that SBEII activity and starch swelling power are positively correlated (*r* = 0.939). In contrast, the increase in swollen pellet volume was strongly but negatively correlated (*r* = −0.976) to the reduction of *T*_o_ (Table 3).

## Discussion

The manipulation of starch properties is important commercially for various industrial applications, as well as for the production of food ingredients. Several loss-of-function mutants and RNAi knockdown studies have helped elucidate the role of particular enzymes in starch biosynthesis [3], but overexpression of starch biosynthetic enzymes is more difficult to accomplish and few examples exist. In one study, overexpression of *SBEIIb* in rice produced starch with increased branching, reduced *T*_o_, increased solubility and increased swelling power [22]. However, potato starch has several advantages over cereal starch for commercial applications. Rice starch granules are small (around 5 μm) and polygonal with sharp edges [29], a high lipid content and a high *T*_o_, whereas potato starch granules are of a large oval shape of average size 40 μm and are less crystalline than rice starch [8,30,31]. Starch from potato is also highly phosphorylated relative to cereal starch [32]. The large granule size, low crystallinity, low lipid content and high phosphate content give potato starch a low temperature for onset of gelatinisation, and a high swelling power and viscosity that are useful for both culinary and industrial applications.

Amylopectin chains have a polymodal size distribution, and models of amylopectin structure propose a long C chain that contains the single reducing terminal residue, long inner B chains that carry additional branches, and shorter, outer A chains that do not carry additional branches and are linked to the B chains [33]. Polymodal chain size distributions of DP 6–12, 13–24, 25–36 and ≥37 are believed to correspond to A chains (DP6–12), and various classes of longer B chains [34]. The abundance of short A chains of DP 6–12 probably determines the starch crystalline allomorph [31]. A range of *SBEII* overexpression levels increased the number of amylopectin chains of DP ≤ 12 and caused a particularly large increase in those of DP6 (Figure 4B). This did not result in any obvious difference in the shape or size of the granules or in their iodine staining properties (Figure 5), presumably because amylose content was not substantially affected (Table 1). Iodine staining is largely due to the content of amylose, which has a 20-fold stronger binding capacity for iodine than does amylopectin [26]. In contrast, knockdown of *GBSS* resulted in a pale purple colour upon iodine staining (Figure 5B), indicating a low amylose content, but with dark blue staining and intense fluorescence at the hilum (Figure 5B,F). A high concentration of amylose at the hilum core even in *GBSS*-silenced lines is consistent with previous observations [6,27]. Knockdown of *GBSS* did not bring about an alteration in the appearance of the granules (Figure 5), [27].

Starch gelatinisation, caused by heating starch in excess water (or exposure to chaotropic agents such as urea), is due to disruption of the molecular order in the crystalline regions and results in irreversible granular swelling and starch solubilisation. The temperature required for starch gelatinisation, and the swelling volume achieved, are affected by granule size and shape, the amylose:amylopectin ratio, amylopectin branching, phosphorus content and other factors. Overexpression of *SBEII* created amylopectin with a high abundance of short DP6 side chains and facilitated gelatinisation, enabling gelatinisation to occur at a lower temperature (Table 3) and at a lower concentration of urea (Figure 7). Granule size and shape were not affected by *GBSS* knockdown or *SBEII* overexpression, but amylose content, amylopectin branching and phosphorus content were altered. Starch phosphorylation occurs mainly on the longer branches of amylopectin, with only a minor proportion on short amylopectin chains, and is essentially absent on amylose [28,35]. Thus, knockdown of *GBSS* expression and the resulting low amylose content had little effect on the degree of starch phosphorylation (Table 2). In *SBEII* overexpression lines, the increased number of short branches of DP ≤ 12 and reduced number of longer branches of DP ≥ 20 should reduce total starch phosphorus content to some extent, and a 25% reduction was observed.

Studies on cereal starch have suggested that swelling power is exclusively due to the properties of the amylopectin, with amylose and lipids inhibiting swelling [36]. In *SBEII*-overexpressing line 1047–17, the swelling volume in 4 M urea was increased to approximately 65% of the maximal volume achieved (in 9 M urea), compared with only 20% for wild-type (Figure 7). A very low amylose content in line 1041–3 changed the gelatinisation response to urea concentration, and increased swelling power at >6 M urea (Figure 7), [17,37], but amylose content in the *SBEII* overexpressing lines was hardly different from wild-type (Table 1). This suggests that amylopectin branching pattern was the major factor influencing starch swelling power. Previous studies have found that *T*_o_ decreased when there was an increased frequency of short amylopectin branches, due either to knockdown of *isoamylase1* or overexpression of *SBEIIb* in rice [22,29], or knockdown of *SSII/SSIII* or overexpression of bacterial glycogen branching enzyme in potato [21,38]. The converse finding, that an increased *T*_p_ results from a reduction in the frequency of short branches due to knockdown of *SBEII* genes in rice and barley [9,10] or the *amylose extender* (knockout of *SBEIIb*) mutant of maize [7], confirms the influence of starch branching frequency on thermal properties.

In lines with a range of *SBEII* expression, there was a correlation between SBEII activity, the degree of reduction in *T*_o_ and the degree of increase in the swelling volume in 4 M urea. In other studies, comparisons between transgenic potato lines found that swelling power was decreased when either amylopectin branching was reduced (knockdown of *SBEI/SBEII*), or phosphorus content was reduced (knockdown of *GWD* (glucan, water dikinase)) [39]. A knockdown of *GWD* that reduced starch C6-linked phosphorus content by 85% had little effect on *T*_o_ or*T*_p_, although swelling power was considerably reduced [39,40]. We observed increased swelling power in starch from our *SBEII* overexpressing lines, despite a 25% reduction in phosphorus content. Thus, increased amylopectin branching rather than small changes in amylose or phosphorus content most likely produced both the lowered *T*_o_ and increased swelling power. Consistent with the data presented here, increased branching in rice starch was also reported to increase swelling volume [22], although differences were not quantified and the swelling power of rice starch is very small relative to that of potato starch [41].

The present study shows that overexpression of potato *SBEII* increases the proportion of short chains of DP6-12, consistent with *in vitro* experiments that found potato SBEII transfers a high number of chains of DP6 [42] and knockdown of *SBEII* that caused an increase in average chain length [14]. We propose that this results in an abnormal amylopectin structure with reduced crystallinity, thus altering gelatinisation properties. In contrast, the increase in *T*_o_ found in the *GBSS* knockdown lines was most likely due to the reduced amylose content, since the degree of amylopectin branching and the phosphorus content were not substantially altered (Figure 4D, Table 2). An increase in the gelatinisation temperature of low-amylose potato starch has been noted previously [37,39].

Both genetic manipulations caused a lowered peak viscosity as determined by RVA, which measures changes in viscosity of the starch as it is heated and cooled under constant stirring (Figure 6). A rapid rise in viscosity upon heating caused by gelatinisation is followed by a decline as the amylopectin matrix structure is broken down by mechanical shearing. The two amylose-containing starches (WT898 and 1047–17) showed a higher peak viscosity and a slightly higher setback viscosity after cooling than line 1041–3. The reduced peak and final viscosities of low-amylose starches, such as that from 1041–3, are consistent with previous studies [15,43]. Starch from a line overexpressing *SBEII* also had a reduced viscosity, presumably due to the increased ratio of short to long amylopectin branches [21]. This is useful commercially since a reduction in the viscosity of potato starch is highly desirable in the production of manufactured products, in order to facilitate handling [37].

## Conclusions

The data presented here show that overexpression of the potato *SBEII* gene can be achieved in potato, and that a relatively small single-intron construct obviates the difficulties of either manipulating a very large genomic fragment or cloning a functional *SBEII* cDNA gene in bacteria. The use of potato rather than cereals is advantageous since transformation is more facile and starch yields are high. The abundance of short amylopectin branches and thermal properties are highly correlated, and elsewhere highly branched starch with many short chains has been shown to have a lower gelatinisation temperature and improved rheological properties [44]. The *T*_o_ of native potato starch is up to 10°C lower than that of rice starch [41], and in the work presented here the increased abundance of short amylopectin branches has lowered it by another 3°C and reduced viscosity while increasing swelling power. Such starches may be expected to find many uses in the food and manufacturing industries.

Additionally, although modifying the amylopectin branching pattern *in planta* requires the use of genetic modification, starch produced in this way may replace chemically modified starch in food applications and thus remove environmental concerns and food additive labelling requirements. Previously, studies of overexpression of starch branching enzymes in potato have used bacterial enzymes [19-21]. Since the transgene used for overexpression in the current work consisted of a modified potato *SBEII* gene that did not contain any non-potato elements and was controlled by a potato promoter and terminator (both from the *GBSS* gene), the approach could be used to create entirely intragenic plants where all the transferred DNA (including T-DNA borders) is derived from the potato genome [45]. The use of intragenic technologies with all-potato DNA may ultimately remove the need for genetic modification labelling [45].

## Methods

### Construct assembly and transgenic plants

It did not prove possible to propagate a complete cDNA of potato *SBEII* in *Escherichia coli*, so an alternative strategy was employed. A suitable short intron to prevent translation of a functional SBEII protein in bacteria was identified at amino acid 439, and primers (Additional file 1: Table S1) were designed to incorporate existing *Afl*II and *Sac*I sites in the potato sequence that flanked the putative intron. PCR amplification from potato (cv ‘Karaka’) gDNA using primers FW3 and RW10 produced a 374 bp fragment that contained an intron of 231 bp.

The upstream portion of potato *SBEII* cDNA was PCR-amplified using primers SBE-A and RW11 (incorporating a *Afl*II site) to give cDNA1, and the downstream portion using primers F4 (incorporating a *Sac*I site) and SBE-B to give cDNA2. All PCR products were verified by DNA sequencing. cDNA1 and the genomic fragment containing the intron were ligated together using the *Afl*II site, then the cDNA1:gDNA fragment (blunt-*Sac*I) and the cDNA2 fragment (*Sac*I-blunt) were ligated in a three-way ligation into expression vector GBSSPTpUC57 (a gift from Dr A. J. Conner), between the *GBSS* promoter and the *GBSS* terminator. This produced a 4.46 kb construct pDB1047 consisting of *GBSS*_*pro*_:cDNA1:gDNA:cDNA2:*GBSS*_*term*_ (Additional file 1: Figure S1).

To produce an inverted repeat construct for silencing of *GBSS*, two fragments of potato *GBSS* cDNA were amplified by PCR using primer pairs GBSS-A and GBSS-B, and GBSS-C and GBSS-D (Additional file 1: Table S1). Primers GBSS-A and GBSS-B amplified a fragment of 1364 bp spanning amino acids 132–585 of the 607-amino acid protein, adding an initiating methionine codon and restriction sites *Xho*I upstream and *Pst*I downstream. Primers GBSS-C and GBSS-D amplified a fragment of 987 bp spanning amino acids 135–463, adding restriction sites *Xba*I and *Stu*I upstream and *Pst*I downstream. The GBSS-A/GBSS-B PCR product was ligated in the sense orientation to the GBSS-C/GBSS-D PCR product in the antisense orientation, using the *Pst*I site. This DNA was ligated into expression vector GBSSPTpUC57 between the *GBSS* promoter and the *GBSS* terminator to produce construct pJZ1041.

Construct pDB1047 was transferred into binary vector pMOA33, conferring resistance to kanamycin, and construct pJZ1041 was transferred into binary vector pMOA34, conferring resistance to hygromycin [46]. These binary vectors were electroporated into *Agrobacterium tumefaciens* LBA4404, which was used to transform *Solanum tuberosum* cv ‘Karaka’, a cultivar common in New Zealand, as described [47]. Plantlets were confirmed as transgenic by screening for the presence of the transgene and the selectable marker gene using PCR (Additional file 1: Figure S2). Empty vector transformants were produced similarly, and non-transformed wild-type (WT) material was regenerated through the tissue culture process (lacking the selectable antibiotic) as controls. Primary transformants were grown and screened as described below.

### Plant growth and molecular analysis

Plantlets were potted in plastic bags (16 L) of potting mix and grown to maturity in a containment glasshouse in Palmerston North (40° 21′ S, 175° 36′ E) during the southern summer and autumn months (January to May). Glasshouse conditions were approximately 15 h daylight per day, with supplementary cooling and heating providing a maximum daytime temperature of 27°C and a minimum night-time temperature of 16°C. The plant aerial portions were allowed to senesce naturally. Three tubers per plant were peeled, chopped into small cubes and frozen in liquid nitrogen before being stored at −80°C for RNA or enzyme assay. RNA was prepared using the hot borate method [48], and 10 μg per lane was separated by electrophoresis in 1.2% agarose denaturing formaldehyde gels. The RNA was blotted to Hybond-XL nylon membrane (GE Healthcare, Little Chalfont, UK) and immobilized by ultraviolet irradiation. Labelled probes were produced using PCR products SBEII cDNA2 and GBSS-A/GBSS-B as templates with [α-^32^P]dATP and the Klenow fragment of DNA polymerase I. RNA gel blots were hybridised overnight with the labelled probes in Church and Gilbert [49] buffer at 65°C, washed in 1x SSC at 65°C and exposed to X-ray film. For analysis of starch physicochemical properties, independent primary transformants were multiplied by excising axillary buds of plantlets in tissue culture and regenerating them to whole plants and tubers in subsequent summers. Biological replicates consisted of pooled tissue of three tubers from three or four different but genetically identical plants.

### Native PAGE determination of SBEII activity

Tuber tissue was powdered in liquid nitrogen, and 100 mg was extracted with 100 μL of extraction buffer (50 mM HEPES-NaOH, pH 7.4, 2 mM MgCl_2_, 50 mM 2-mercaptoethanol, 12.5% (v/v) glycerol), incubated on ice for 5 min then centrifuged twice at 16,000 × *g* for 15 min [17]. Soluble proteins in the supernatant were used as the crude enzyme preparation. Proteins (150 μg per lane) were separated on native 5% (w/v) polyacrylamide gels containing substrates for SBE activity as described by Tetlow *et al.* [24]. After electrophoresis, gels were rinsed, incubated in reaction buffer at 30°C for 2 h then stained with I_2_/KI as described by Nishi *et al.* [17]. Gels were photographed immediately after staining. Gel pictures were converted to greyscale, and relative density in the SBEII spots quantified using MultiGauge software (Fujifilm, Tokyo, Japan).

### Starch preparation

Starch was prepared by chopping peeled fresh tubers into small pieces, adding an equal amount of ice-cold water and crushing the tissue with a potato masher. Starch granules were washed through a 200 μm mesh sieve, and the suspension allowed to settle overnight. The supernatant and any discoloured layer above the white starch pellet were discarded. The starch was resuspended in a large volume of ice-cold water and the settling step repeated. The pellet of starch granules was freeze-dried and used for analysis.

### Microscopy

Aqueous, unstained suspensions of starch grains were observed by brightfield, differential interference contrast (DIC) and simple polarising microscopy (crossed polars but no phase plate) using an Olympus Vanox AHT3 microscope (Olympus Optical Co Ltd., Tokyo, Japan). Brightfield microscopy was also used to view starch stained with iodine using a stain solution of 0.2% I_2_ in a 2% aqueous solution of KI at a dilution ratio of 1:100 stain:starch suspension. For scanning electron microscopy (SEM), dry starch powder was lightly sprinkled onto carbon adhesive tabs attached to aluminium stubs and was viewed uncoated in a FEI Quanta 250 SEM (FEI Company, Hillsboro, OR, USA) using variable pressure mode to reduce charging (chamber pressure 60 Pa, accelerating voltage 5 kV).

The reducing ends of starch molecules were fluorescently labelled within the starch granules using APTS as described by Blennow *et al.* [27]. Optical sections were taken under a Leica SP5 DM6000B laser scanning confocal microscope, using an excitation wavelength of 488 nm and detection at the interval from 500 to 535 nm.

### Size exclusion chromatography

Aliquots of starch were suspended in 2 M NaOH, gelatinised at 100°C then dissolved in water as described by Blennow *et al.* [26]. The solubilised starch was applied to a Waters Ultrahydrogel Linear column (300 × 7.8 mm) with an exclusion limit of 7 × 10^6^ Da, and eluted with 10 mM NaOH at 50°C. Carbohydrate content in the effluent was recorded on-line by measuring refractive index (Waters 2414 Refractive Index detector (Waters Corp, MA, USA)). A large peak at the end of the elution profiles (due to NaOH) was omitted.

### Starch composition

The percentage of amylose present in the starch was measured using the Megazyme K_AMYL Amylose kit (Megazyme, Bray, Ireland). Starch branching patterns were assessed using a micro-scale FACE procedure to measure chain length size distribution [50]. The degree of C6 and C3 starch phosphorylation was determined using a mass spectrometric method [51].

### Starch properties

Thermal analysis of gelatinisation properties was performed using a Q2000 differential scanning calorimeter (DSC) (TA Instruments, New Castle, DE, USA). Starch (~5 mg) was accurately weighed into Tzero aluminium hermetic pans (TA Instruments), and 2.33 μL water mg^−1^ starch added to give a suspension containing 70% water. The pans were hermetically sealed and left at room temperature (20°C) for at least 2 h. DSC conditions were: equilibration at 10°C for 5 min, then heating at 5°C min^−1^ to 100°C. The heat flow curves were analysed using TA Universal Analysis 2000 software version 4.5A build 4.5.0.5 using the “integrate peak sigmoid horizontal” option over the range 50–80°C. Variables calculated were peak onset temperature (*T*_o_), peak maximum temperature (*T*_p_), peak conclusion temperature (*T*_c_) and peak area (Δ*H*), which provides an estimate of enthalpy in J g^−1^.

Starch pasting properties were evaluated using a rapid visco analyser (RVA) (Newport Scientific, Sydney, Australia). Approximately 1 g of starch was accurately weighed and dried at 130°C to constant weight. A starch suspension was prepared at 7% (w/w) on a dry weight basis in distilled water, with a total weight of 28 g. The suspension was equilibrated at 50°C for 1 min, heated to 95°C at 6°C min^−1^, maintained at 95°C for 5 min, then cooled to 50°C at 6°C min^−1^, with constant stirring at 160 rpm.

The swelling volume of starch was determined using a urea procedure [17]. Freeze-dried starch (20 mg) was mixed with 1 mL of urea solution (0 to 9 M). The mixtures were allowed to stand at room temperature (23°C) for 24 h, centrifuged at 8000 × *g* for 20 min, then allowed to stand at room temperature for 1 h. The volumes of the swollen pellet were determined by subtracting the volume of the supernatant from the original 1 mL volume.

### Statistical analysis

Means were compared using one-way ANOVA. Least significant differences (LSDs) were used to establish which means differed significantly (*P* = 0.05). Since for SBEII activity the data was relative and was scaled so that wild-type was 1, the wild-type data was not included in the ANOVA; pooled standard errors from the ANOVA were used in one-sample t-tests to test whether each mean was significantly different from 1. Correlation coefficients were calculated between means. Analysis was carried out using Genstat (version 17, 2014, VSNI Ltd, Hemel Hempstead, UK).

## Additional file

Additional file 1: Table S1. Primers used for PCR amplification. **Figure S1.** Sequence of the hybrid cDNA/gDNA SBEII-intron construct. **Figure S2.** PCR screening of transgenic plants for the presence of the transgene and the selectable marker gene. **Figure S3.** RNA gel blot analysis of *SSII* and *SSIII* mRNA abundance in tubers from plants overexpressing *SBEII*.

## Abbreviations

APTS: 8-amino-1,3,6-pyrenetrisulfonic acid
DP: Degree of polymerisation
DSC: Differential scanning calorimeter
FACE: Fluorophore-assisted carbohydrate electrophoresis
GBSS: Granule-bound starch synthase
PAGE: Polyacrylamide gel electrophoresis
RVA: Rapid visco analyser
SBE: Starch branching enzyme
SS: Starch synthase
