# Supplementary material for: Overexpression of STARCH BRANCHING ENZYME II increases short-chain branching of amylopectin and alters the physicochemical properties of starch from potato tuber
Source: BMC Biotechnol. 2015 Apr 29;15:28. doi: 10.1186/s12896-015-0143-y (PMC4414359; doi:10.1186/s12896-015-0143-y)
Supplement: Additional file 1: Table S1. — Primers used for PCR amplification. Figure S1. Sequence of the hybrid cDNA/gDNA SBEII-intron construct. Figure S2. PCR screening of transgenic plants for the presence of the transgene and the selectable marker gene. Figure S3. RNA gel blot analysis of SSII and SSIII mRNA abundance in tubers from plants overexpressing SBEII. [file 12896_2015_143_MOESM1_ESM.pdf]

Supplementary Data

Overexpression of *STARCH BRANCHING ENZYME II* increases short-chain branching of amylopectin and alters the physicochemical properties of starch from potato tuber

David A. Brummell, Lyn M. Watson, Jun Zhou, Marian J. McKenzie, Ian C. Hallett, Lyall Simmons, Margaret Carpenter, Gail M. Timmerman-Vaughan

Table S1. Primers used for PCR amplification.

Name	Sequence (5′-3′)
FW3	CCGACGACCTTAAGTCTTTG
RW10	CAATGATAACCACGAGCTCC
SBE-A	AAAACCATGGTGTATACACTCTCTGGAGTTCG
RW11	CAAAGACTTAAGGTCGTCGGG
F4	GGAGCTCGTGGTTATCATTGG
SBE-B	AAAACTCGAGCAACGCGATCACAAGTTCG
GBSS-A	AAACTCGAGGCCTAGGATACTAGCGTTGCGGTTGAG
GBSS-B	AAACTGCAGAAGCTCCTAAGCCCAATAGC
GBSS-C	AAATCTAGAGGCCTGTTGCGGTTGAGGTCAAAG
GBSS-D	TTTCTGCAGACATTGAATTTGCCACTCC

```
NcoI
CCATGGTGTATACACTCTCTGGAGTTCGTTTTCCTACTGTTCCATCAGTGTACAAATCTAATGGATTCAGCAGTAATGGTGATCGGAGGAATGCTAATATTTCTGTATTCTTGAAAAAA
 ▶ M  V  Y  T  L  S  G  V  R  F  P  T  V  P  S  V  Y  K  S  N  G  F  S  S  N  G  D  R  R  N  A  N  I  S  V  F  L  K  K
        BglII
CACTCTCTTTCACGGAAGATCTTGGCTGAAAAGTCTTCTTACAATTCCGAATCCCGACCTTCTACAATTGCAGCATCGGGGAAAGTCCTTGTGCCTGGAATCCAGAGTGATAGCTCCTC
▶ H  S  L  S  R  K  I  L  A  E  K  S  S  Y  N  S  E  S  R  P  S  T  I  A  A  S  G  K  V  L  V  P  G  I  Q  S  D  S  S  S
                                                                                          NheI
ATCCTCAACAGATCAATTTGAGTTCGCTGAGACATCTCCAGAAAATTCCCCAGCATCAACTGATGTAGATAGTTCAACAATGGAACACGCTAGCCAGATTAAAACTGAGAACGATGACG
▶  S  S  T  D  Q  F  E  F  A  E  T  S  P  E  N  S  P  A  S  T  D  V  D  S  S  T  M  E  H  A  S  Q  I  K  T  E  N  D  D
TTGAGCCGTCAAGTGATCTTACAGGAAGTGTTGAAGAGCTGGATTTTGCTTCATCACTACAACTACAAGAAGGTGGTAAACTGGAGGAGTCTAAAACATTAAATACTTCTGAAGAGACA
▶V  E  P  S  S  D  L  T  G  S  V  E  E  L  D  F  A  S  S  L  Q  L  Q  E  G  G  K  L  E  E  S  K  T  L  N  T  S  E  E  T
                                                                                                   HincII
ATTATTGATGAATCTGATAGGATCAGAGAGAGGGGCATCCCTCCACCTGGACTTGGTCAGAAGATTTATGAAATAGACCCCCTTTTGACAAACTATCGTCAACACCTTGATTACAGGTA
▶ I  I  D  E  S  D  R  I  R  E  R  G  I  P  P  P  G  L  G  Q  K  I  Y  E  I  D  P  L  L  T  N  Y  R  Q  H  L  D  Y  R  Y
                                                       HindIII
TTCACAGTACAAGAAACTGAGGGAGGCAATTGACAAGTATGAGGGTGGTTTGGAAGCTTTTTCTCGTGGTTATGAAAGAATGGGTTTCACTCGTAGTGCTACAGGTATCACTTACCGTG
▶  S  Q  Y  K  K  L  R  E  A  I  D  K  Y  E  G  G  L  E  A  F  S  R  G  Y  E  R  M  G  F  T  R  S  A  T  G  I  T  Y  R
                     PvuII
AGTGGGCTCCTGGTGCCCAGTCAGCTGCCCTCATTGGGGATTTCAACAATTGGGACGCAAATGCTGACTTTATGACTCGGAATGAATTTGGTGTCTGGGAGATTTTTCTGCCAAATAAT
▶E  W  A  P  G  A  Q  S  A  A  L  I  G  D  F  N  N  W  D  A  N  A  D  F  M  T  R  N  E  F  G  V  W  E  I  F  L  P  N  N
GTGGATGGTTCTCCTGCAATTCCTCATGGGTCCAGAGTGAAGATACGTATGGACACTCCATCAGGTGTTAAGGATTCCATTCCTGCTTGGATCAACTACTCTTTACAGCTTCCTGATGA
▶ V  D  G  S  P  A  I  P  H  G  S  R  V  K  I  R  M  D  T  P  S  G  V  K  D  S  I  P  A  W  I  N  Y  S  L  Q  L  P  D  E
AATTCCATATAATGGAATATATTATGATCCACCCGAAGAGGAGAGGTATATCTTCCAACACCCACGGCCAAAGAAACCAAAGTCGGTGAGAATATATGAATCTCATATTGGAATGAGTA
▶  I  P  Y  N  G  I  Y  Y  D  P  P  E  E  E  R  Y  I  F  Q  H  P  R  P  K  K  P  K  S  V  R  I  Y  E  S  H  I  G  M  S
 BspEI                                                     HindIII
GTCCGGAGCCTAAAATTAACTCATACGTGAATTTTAGAGATGAAGTTCTTCCTCGCATAAAAAAGCTTGGGTACAATGCGGTGCAAATTATGGCTATTCAAGAGCATTCTTATTATGCT
▶S  P  E  P  K  I  N  S  Y  V  N  F  R  D  E  V  L  P  R  I  K  K  L  G  Y  N  A  V  Q  I  M  A  I  Q  E  H  S  Y  Y  A
                                                             AflII
AGTTTTGGTTATCATGTCACAAATTTTTTTGCACCAAGCAGCCGTTTTGGAACGCCCGACGACCTTAAGTCTTTGATTGATAAAGCTCATGAGCTAGGAATTGTTGTTCTCATGGACAT
▶ S  F  G  Y  H  V  T  N  F  F  A  P  S  S  R  F  G  T  P  D  D  L  K  S  L  I  D  K  A  H  E  L  G  I  V  V  L  M  D  I
                                                                                             HincII
TGTTCACAGgtattaattaatcttttactattgcaactgtgtttagaaaagctatggatggggtttttgttgtcatttcttgtttttgattgttgacgtctaattcataaatgaaagaa
▶  V  H  R
              XbaI
atggtgtttcattctagaatctaatcataataatggttagcctttttttttttactggcacttattttctattgacatgctttactttgtccttaactcactggaattatgcaacctttt
                                                                                        SacI
tgCAGCCATGCATCAAATAATACTTTAGATGGACTGAACATGTTTGACGGCACAGATAGTTGTTACTTTCACTCTGGAGCTCGTGGTTATCATTGGATGTGGGATTcCCGCCTCTTTAA
  ▶ S  H  A  S  N  N  T  L  D  G  L  N  M  F  D  G  T  D  S  C  Y  F  H  S  G  A  R  G  Y  H  W  M  W  D  S  R  L  F  N
CTATGGAAACTGGGAGGTACTTAGGTATCTTCTCTCAAcTGCGAGATGGTGGTTGGATGAGTTCAAATTTGATGGATTTAGATTTGATGGTGTGACATCAATGATGTaTgCTCACCACG
▶  Y  G  N  W  E  V  L  R  Y  L  L  S  T  A  R  W  W  L  D  E  F  K  F  D  G  F  R  F  D  G  V  T  S  M  M  Y  A  H  H
                                                                                    HincII
GATTATCGGTGGGATTCACTGGGAACTACGAGGAATACTTTGGACTCGCAACTGATGTGGATGCTGTTGTGTATCTGATGCTGGTCAACGATCTTATTCATGGGCTTTTCCCAGATGCA
▶G  L  S  V  G  F  T  G  N  Y  E  E  Y  F  G  L  A  T  D  V  D  A  V  V  Y  L  M  L  V  N  D  L  I  H  G  L  F  P  D  A
                                                                                     NdeI
ATTACCATTGGTGAAGATGTTAGCGGAATGCCGACATTTTGTaTTCCCGTTCAAGATGGGGGTGTTGGCTTTGACTATCGGCTGCATATGGCAATTGCTGATAAATGGATTGAGTTGCT
▶ I  T  I  G  E  D  V  S  G  M  P  T  F  C  I  P  V  Q  D  G  G  V  G  F  D  Y  R  L  H  M  A  I  A  D  K  W  I  E  L  L
CAAGAAACGGGATGAGGATTGGAGAGTGGGTGATATTGTTCATACACTGACAAATAGAAGATGGTCGGAAAAGTGTGTTTCATACGCTGAAAGTCATGATCAAGCTCTAGTCGGTGATA
▶  K  K  R  D  E  D  W  R  V  G  D  I  V  H  T  L  T  N  R  R  W  S  E  K  C  V  S  Y  A  E  S  H  D  Q  A  L  V  G  D
                                                              HincII
AAACTATAGCATTCTGGCTGATGGACAAGGATATGTATGATTTTATGGCTCTGGATAGACCGTCAACATCATTAATAGATCGTGGGATAGCATTGCACAAGATGATTAGGCTTGTAACT
▶K  T  I  A  F  W  L  M  D  K  D  M  Y  D  F  M  A  L  D  R  P  S  T  S  L  I  D  R  G  I  A  L  H  K  M  I  R  L  V  T
                  KpnI                 EcoRI                        AvrII
ATGGGATTAGGAGGgGAAGGGTACCTAAATTTCATGGGAAATGAATTCGGCCACCCTGAGTGGATTGATTTCCCTAGGGCTGAACAACACCTCTCTGATGGCTCAGTAATTCCCgGAAA
▶ M  G  L  G  G  E  G  Y  L  N  F  M  G  N  E  F  G  H  P  E  W  I  D  F  P  R  A  E  Q  H  L  S  D  G  S  V  I  P  G  N
CCAATTCAGTTATGATAAATGCAGACGGAGATTTGACCTGGGAGATGCAGAATATTTAAGATACCGTGGGTTGCAAGAATTTGACCGGGCTATGCAGTATCTTGAAGATAAATATGAGT
▶  Q  F  S  Y  D  K  C  R  R  R  F  D  L  G  D  A  E  Y  L  R  Y  R  G  L  Q  E  F  D  R  A  M  Q  Y  L  E  D  K  Y  E
TTATGACTTCAGAACACCAGTTCATATCACGAAAGaATGAAGGAGATAGGATGATTGTATTTGAAAgAGGAAACCTAGTTTTTGTCTTTAATTTTCACTGGACAAAAaGCTATTCAGAC
▶F  M  T  S  E  H  Q  F  I  S  R  K  N  E  G  D  R  M  I  V  F  E  R  G  N  L  V  F  V  F  N  F  H  W  T  K  S  Y  S  D
TATCGCATAGGCTGCCTGAAGCCTGGAAAATACAAGGTTGCCTTGGACTCAGATGATCCACTTTTTGGTGGCTTCGGGAGAATTGATCATAATGCCGAATATTTCACCTTTGAAGGATG
▶ Y  R  I  G  C  L  K  P  G  K  Y  K  V  A  L  D  S  D  D  P  L  F  G  G  F  G  R  I  D  H  N  A  E  Y  F  T  F  E  G  W
                                                             SpeI
GTATGATGATCGTCCTCGTTCAATTATGGTGTATGCACCTAGTAGAACAGCAGTGGTCTATGCACTAGTAGACAAAGAAGAAGAAGAAGAAGAAGAAGTAGCAGTAGTAGAAGAAGTAG
▶  Y  D  D  R  P  R  S  I  M  V  Y  A  P  S  R  T  A  V  V  Y  A  L  V  D  K  E  E  E  E  E  E  E  V  A  V  V  E  E  V
                                         junction marker (2898)
TAGTAGAAGAAGAATGAACGAACTTGTGATCGCGTTGCtcga
▶V  V  E  E  E  •
```

Figure S1. Sequence of the hybrid cDNA/gDNA SBEII-intron construct. PCR fragments consisting of an upstream region of the coding sequence amplified from cDNA (dark blue), a central coding region amplified from genomic DNA (light blue) interrupted by an intron (black, lower case), and a downstream region of the coding sequence amplified from cDNA (magenta) were ligated together using internal *Afl*II and *Sac*I restriction sites. The sequence was inserted into an expression vector between the *GBSS* promoter and *GBSS* terminator.

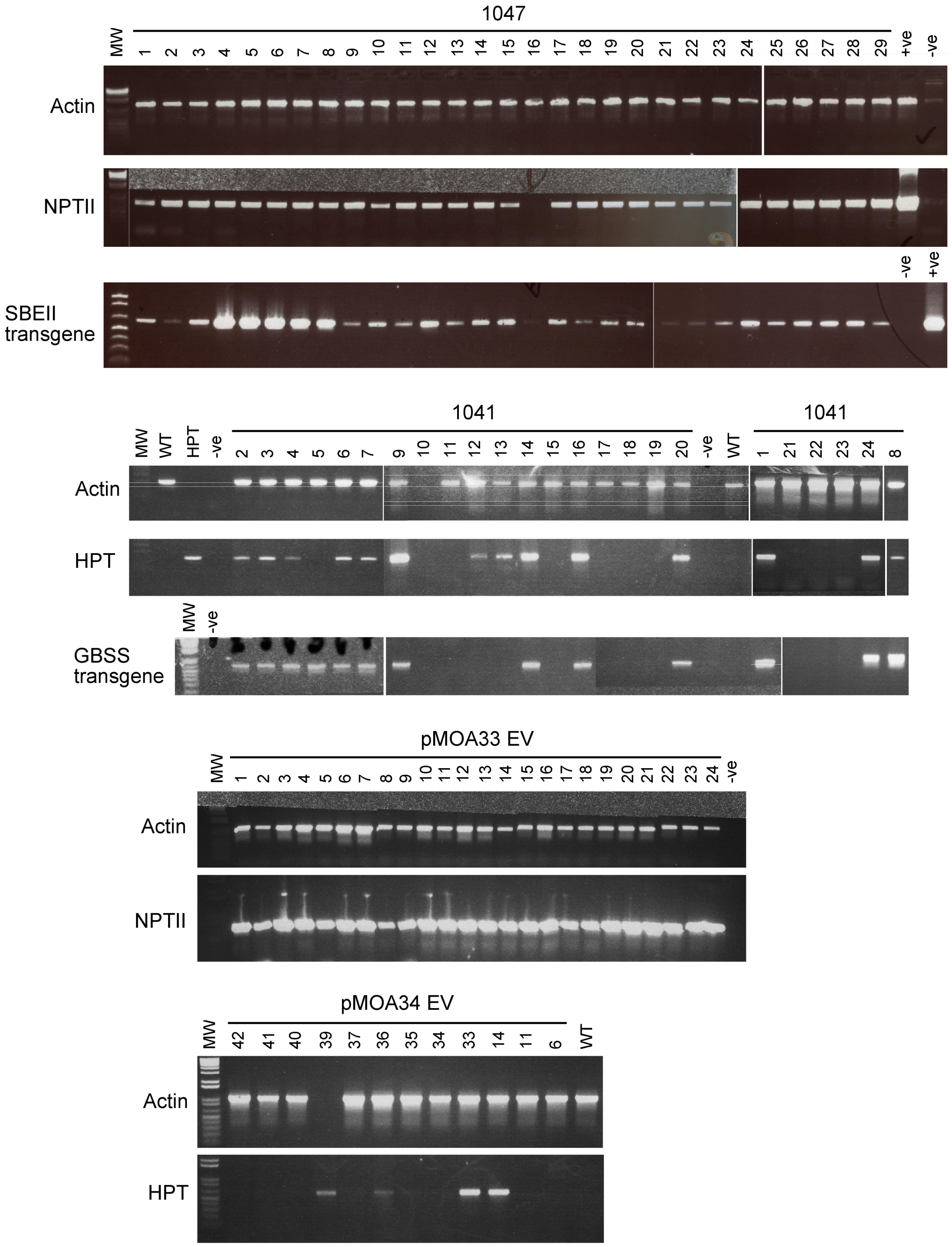

Figure S2. PCR screening of genomic DNA from primary transformants for the presence of the selectable marker gene and the transgene. The primers for the *SBEII-intron* transgene (construct 1047) spanned the *SBEII-GBSS$_{term}$* junction. The primers for the *GBSS* RNAi transgene (construct 1041) spanned the junction between the antisense arm of the inverted repeat and the *GBSS$_{term}$*. Empty vector (EV) control transformants were screened for the presence of the selectable marker gene only.

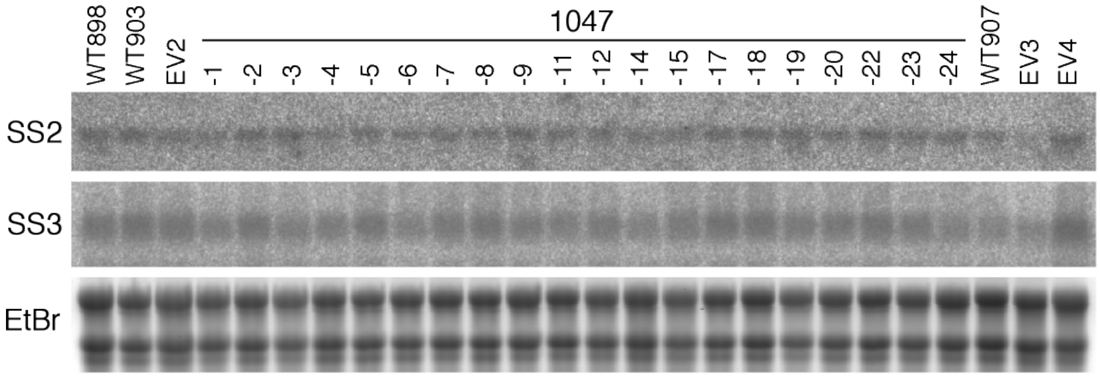

Figure S3. RNA gel blot analysis showing mRNA abundance of *STARCH SYNTHASE II* (SS2) and *STARCH SYNTHASE III* (SS3) in tubers from the 1047 population of plants, overexpressing *STARCH BRANCHING ENZYME II*. Wild-type (WT) and empty vector (EV) controls are included for comparison. Equivalency of RNA loading (10 µg per lane) was assessed by staining with ethidium bromide (EtBr).
